# CRISPR/Cas12a-mediated labeling of MET receptor enables quantitative single-molecule imaging of endogenous protein organization and dynamics

**DOI:** 10.1016/j.isci.2020.101895

**Published:** 2020-12-07

**Authors:** Tim N. Baldering, Christos Karathanasis, Marie-Lena I.E. Harwardt, Petra Freund, Matthias Meurer, Johanna V. Rahm, Michael Knop, Marina S. Dietz, Mike Heilemann

**Affiliations:** 1 Single Molecule Biophysics, Institute of Physical and Theoretical Chemistry, Goethe University Frankfurt, Max-von-Laue Str. 7, 60438 Frankfurt, Germany; 2 Center for Molecular Biology of Heidelberg University (ZMBH), 69120 Heidelberg, Germany; 3 German Cancer Research Center (DKFZ), 69120 Heidelberg, Germany

**Keywords:** Biochemistry, Molecular Biology, Biophysics, Biological Sciences Research Methodologies

## Abstract

Single-molecule localization microscopy (SMLM) reports on protein organization in cells with near-molecular resolution and in combination with stoichiometric labeling enables protein counting. Fluorescent proteins allow stoichiometric labeling of cellular proteins; however, most methods either lead to overexpression or are complex and time demanding. We introduce CRISPR/Cas12a for simple and efficient tagging of endogenous proteins with a photoactivatable protein for quantitative SMLM and single-particle tracking. We constructed a HEK293T cell line with the receptor tyrosine kinase MET tagged with mEos4b and demonstrate full functionality. We determine the oligomeric state of MET with quantitative SMLM and find a reorganization from monomeric to dimeric MET upon ligand stimulation. In addition, we measured the mobility of single MET receptors *in vivo* in resting and ligand-treated cells. The combination of CRISPR/Cas12a-assisted endogenous protein labeling and super-resolution microscopy represents a powerful tool for cell biological research with molecular resolution.

## Highlights

• CRISPR/Cas12a enables endogenous protein labeling for super-resolution microscopy
• HEK293T cells were generated with MET endogenously labeled with mEos4b
• Quantitative PALM microscopy reports efficient dimerization of MET receptor
• Single-particle tracking shows increased MET immobilization upon ligand treatment

## Graphical Abstract

**Figure:**
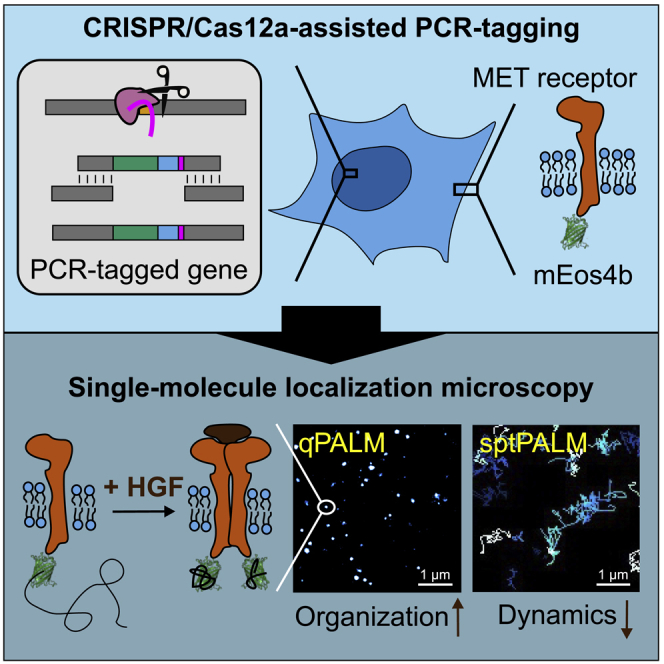

## Introduction

Super-resolution microscopy enables the investigation of protein organization and dynamics at the nanoscale and opens the door for a molecular view on protein function in cells (Schermelleh et al., 2019). Single-molecule localization microscopy (SMLM) is a super-resolution technique that generates images from stochastic activation and detection of single fluorescent emitters (Sauer and Heilemann, 2017). SMLM can report on molecule numbers, simultaneously providing access to quantitative information on how proteins assemble in a cell (Dietz and Heilemann, 2019). Photoactivated localization microscopy (PALM) is a variant of SMLM that uses photoactivatable or photoconvertible fluorescent proteins that are activated stochastically by irradiation with violet light (Betzig et al., 2006). Fluorescent proteins allow stoichiometric labeling by genetic coupling to a target protein, which avoids unspecific labeling as it can occur with fluorophore-labeled antibodies. In addition, fluorescent proteins allow targeting intracellular proteins and protein domains without the need of cell membrane permeabilization which may induce damage to cellular structures (Whelan and Bell, 2015).

Several options of tagging target proteins with fluorescent proteins are available. Plasmids encoding fusion proteins can be produced synthetically in a short time and introduced into mammalian cells, e.g., by transfection. Transfected cells typically show a range of protein expression levels, which is particularly interesting for the analysis of diseases such as cancer that are associated with overexpression. However, if healthy conditions are to be studied, an endogenous expression level of the protein of interest is required (Lisenbee et al., 2003; Doyon et al., 2011; Gibson et al., 2013). For this purpose, For this purpose, a fluorophore label is favored that assures an endogenous expression level of the labeled protein in the cell.

The emergence of CRISPR/Cas as a genetic engineering tool enabled stoichiometric labeling of endogenous proteins (Deltcheva et al., 2011; Le Cong et al., 2013; Ran et al., 2013; Ratz et al., 2015). Genetic insertions of, e.g., fluorescent proteins by CRISPR/Cas9 can be performed by transfecting cells with a plasmid that codes for the Cas9 enzyme, the crRNA, and the tracrRNA. In addition, a homology template containing the DNA sequence of the fluorescent tag and homology sequences of the gene of interest are required. Using this workflow, CRISPR/Cas9 was used for protein labeling in combination with super-resolution microscopy of mostly intracellular proteins (Ratz et al., 2015; Cho et al., 2016; Hansen et al., 2017; Khan et al., 2017, 2019). However, all these studies use Cas9 for genetic editing and require time-consuming preparation of homology templates. Recently, the enzyme Cas12a was discovered, which has several advantages over Cas9. Cas12a has a higher specificity *in vivo* (Kleinstiver et al., 2016; Kim et al., 2017) and a simpler crRNA structure in comparison to Cas9 (Zetsche et al., 2015). Additionally, Cas12a cuts at some distance from the recognition sequence, resulting in more frequent cuts as the recognition sequence is not destroyed (Zetsche et al., 2015; Moreno-Mateos et al., 2017). Despite numerous advantages of CRISPR/Cas (Yamamoto and Gerbi, 2018), genetic insertions with standard CRISPR/Cas methods are time intense. Taking advantage of the CRISPR/Cas12a system, a new genome engineering approach was introduced (termed termed polymerase-chain reaction [PCR] tagging) which represents a time-saving method to generate the homologous template required for gene insertion (Füller et al., 2020). The main advantage of this technique is the fast generation of the homology template (PCR cassette) by a single PCR reaction using primers (which provide the homology arms) that can be designed with an online tool (www.pcr-tagging.com). Compared to Cas9, the Cas12a enzyme requires only one crRNA that is expressed from a gene located on the PCR cassette. Generation of a gene-specific PCR cassette (by PCR) is thus quick and it can then directly be transfected into cells together with a Cas12a expression plasmid. The crRNA is expressed from the PCR cassette and can thus together with the Cas12a enzyme produce the site-specific DNA cut. The DNA cut is then repaired by homology directed repair of the PCR cassette, which labels the protein of interest with the desired tag. This makes PCR tagging a simple and time-efficient alternative for gene labeling with which high labeling efficiencies are achieved (Füller et al., 2020).

Here, we employ PCR tagging and demonstrate almost complete labeling of the receptor tyrosine kinase MET with the photoconvertible fluorescent protein mEos4b (Paez-Segala et al., 2015) in HEK293T cells. We confirmed the specific integration and functionality of the PCR-tagged MET-mEos4b fusion protein by biochemical methods. Using quantitative super-resolution microscopy, we show that resting MET is largely present as a monomer, whereas in cells treated with the ligands hepatocyte growth factor (HGF) or internalin B (InlB), MET dimers are found. In addition, we performed live-cell single-particle tracking (SPT) experiments of MET-mEos4b and analyzed receptor mobility in untreated cells and upon treatment with HGF.

In summary, we propose PCR tagging as a suitable method for endogenous, stoichiometric, and efficient protein labeling, which in combination with super-resolution microscopy and SPT allows for nanoscopic spatial resolution, quantitative information on protein assemblies, and a native view on protein dynamics.

## Results

### Endogenous tagging of the MET receptor for single-molecule super-resolution microscopy

**Figure 1:**
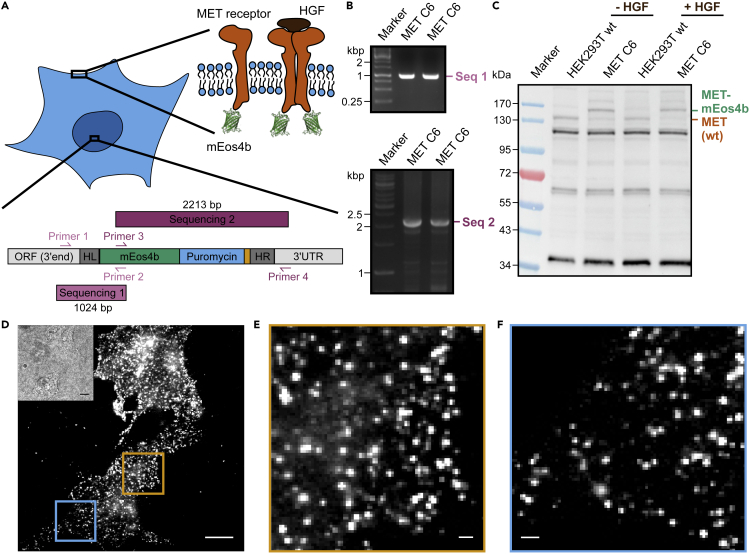
Endogenous tagging of the MET receptor with mEos4b in HEK293T cells (A) Scheme of the generated MET-mEos4b stable cell line showing both the inserted DNA sequence as well as the expressed fusion protein. The inserted DNA fragment as well as the primers that amplify sequences 1 and 2 and whose PCR product includes the inserted fragment as well as a region outside the homology arms (HL and HR, respectively) of the PCR cassette are shown. (B) Agarose gel of the amplified PCR products generated with the primers shown in (A). The markers indicate the size of the amplified DNA products. (C) Western blot with an antibody against the MET receptor in wild-type and PCR-tagged cells with and without the addition of HGF. (D) Standard deviation image of a PALM movie of MET-mEos4b cells. The brightfield image is shown at the upper left. Scale bar represents 10 μm. (E and F) Magnified view of the regions highlighted in the standard deviation image shown in (D) of MET-mEos4b cells. Scale bars of 1 μm. See also Figure S1.

A recent study introduced PCR tagging as a simple and time-efficient method for genetic labeling of target proteins (Füller et al., 2020), which makes it a promising labeling tool for quantitative super-resolution microscopy. Using PCR tagging, we generated a stable cell line in which mEos4b was fused C-terminally to the endogenous MET receptor (Figure 1A). We designed two primers to generate a PCR cassette that is specific for the MET gene and transfected HEK293T cells with the PCR cassette and the Cas12a helper plasmid. After selection of CRISPR/Cas12a-tagged cells via puromycin, individual clones were isolated and verified by fluorescence signals. Pronounced fluorescence was observed in 10 of 16 clones tested. These 10 fluorescent clones were divided into five clones with very high fluorescence and five clones with moderate fluorescence after irradiation with 405 nm. A clone with high fluorescence and a clone with moderate fluorescence were randomly selected and further analyzed by two PCRs that amplified DNA sequences at both ends of the inserted region (outside of the homology arms of the PCR cassette) (Figure 1A). DNA fragments of the expected size were found in both PCRs in one clone showing a moderate fluorescence (Figure 1B; MET C6; duplicate), while the other clone (high fluorescence) was positive in only one PCR (data not shown). Sequencing of the purified PCR fragment of MET C6 cells confirmed the correct insertion of mEos4b, hereafter referred to as MET-mEos4b.

Multiple copies of the chromosome in polyploid cells can lead to a mixed population of tagged and untagged receptors, which decreases the labeling efficiency if not all chromosomal sites are targeted. HEK293T cells contain two or more copies of each chromosome (in total 64–70 chromosomes (Stepanenko et al., 2015)), which is still a moderate number compared to other cell lines (e.g., HeLa, 76–80 (Macville et al., 1999)). We determined the labeling efficiency in the MET-mEos4b cell line from a western blot and found a shift of the MET receptor band of about 25–30 kDa for the stable cell line compared to wild-type MET in HEK293T cells (Figure 1C). This shift in protein size reflects the fusion of the fluorescent protein mEos4b to the MET receptor. In order to determine the fraction of untagged versus tagged receptors in the MET-mEos4b clone, we quantified the band intensities at about 140 kDa and 170 kDa and found that the MET-mEos4b cell line contains approximately 81% mEos4b-tagged MET receptors and 19% of unlabeled MET.

Next, we verified that MET-mEos4b is active by stimulating cells with the physiological ligand of MET, HGF, as well as with a fluorophore-labeled variant of the bacterial ligand InlB, InlB_321_-ATTO 647N, which also targets the MET receptor and was characterized in earlier studies by single-molecule methods (Dietz et al., 2013, 2019; Harwardt et al., 2017). In western blots, MET-mEos4b was phosphorylated upon stimulation with both, HGF and InlB_321_, while untreated cells did not result in phosphorylation of the MET receptor (Figure S1A). Since phosphorylation alone might not necessarily lead to a cellular response (Liang et al., 2018), functional downstream signaling upon stimulation was verified by analyzing the phosphorylation of mitogen-activated protein kinase (Figure S1B). Together, these results demonstrate the native behavior and the same activation pattern of MET-mEos4b compared to wild-type MET receptor.

Upon the verification of efficient labeling and functionality of MET, the next step was to investigate its correct localization at the cell membrane. For that, we performed PALM measurements of HEK293T wild-type cells and the stable cell line expressing MET-mEos4b. While wild-type cells showed only unspecific signal, cells expressing MET-mEos4b showed a clear punctate fluorescence signal at the basal cell membrane with total internal reflection illumination (Figures 1D–1F). We determined the localization precision of MET-mEos4b using a nearest neighbor analysis (Endesfelder et al., 2014) and obtained an average value of 10 ± 3 nm. Furthermore, we addressed the cell-to-cell heterogeneity of MET-mEos4b cells. We determined the number of nanoclusters per μm^2^ and found only small variations between single cells (0.63 ± 0.32 cluster/μm^2^). In summary, these data demonstrate that we have generated a cell line that expresses fully active MET-mEos4b at an expression level of the wild-type MET receptor.

### Changes in oligomerization of endogenous MET after receptor activation

Stoichiometric and efficient labeling is particularly important for quantitative imaging of proteins with microscopy. We were explicitly interested to explore the potential of PCR tagging in quantitative super-resolution microscopy. Quantitative SMLM demands for high-efficient stoichiometric labeling and provides information on protein organization within nanoclusters in cells that is otherwise inaccessible (Krüger et al., 2017; Karathanasis et al., 2020; Möller et al., 2020). These nanoclusters consist of one or more proteins that cannot be resolved as individual units because of a protein packing density within these nanoclusters that is beyond the resolution capabilities of super-resolution PALM. However, the quantitative analysis of fluorescent protein emission kinetics allows to extract the oligomeric state of proteins within these nanoclusters (Dietz and Heilemann, 2019). Here, we used quantitative PALM (qPALM) (Fricke et al., 2015; Hummer et al., 2016; Baldering et al., 2019a) and aimed to determine the oligomeric state of MET-mEos4b before and after ligand stimulation.

**Figure 2:**
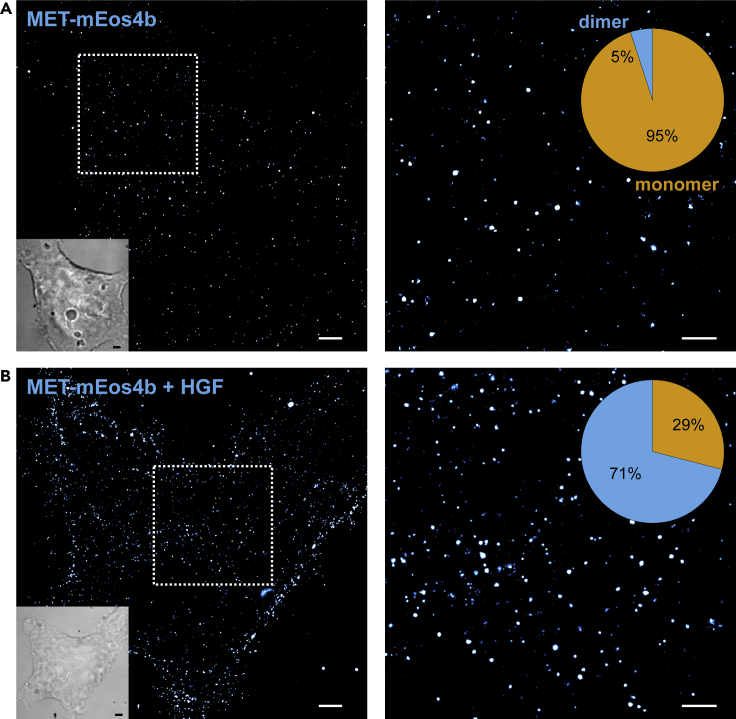
Quantitative PALM analysis of ligand-stimulated and resting MET-mEos4b receptors qPALM images (left side) of resting (A) and HGF-activated MET-mEos4b cells (B). Zoom-ins of regions marked in the left images are shown (right side) with monomer/dimer ratios depicted as pie charts. Scale bars represent 2 µm and 1 µm. See also Figures S2 and S3.

In order to determine whether mEos4b is suitable for qPALM analysis, we first imaged single mEos4b immobilized on a glass surface and analyzed the bleaching probability (p-value) from the single-molecule “blinking” statistics (Figures S2A and S2B). We found a p-value of 0.34 ± 0.01, which is similar to the p-value of mEos3.2 (0.32) (Baldering et al., 2019b), thus showing that mEos4b is suitable for qPALM (Figure S2C). Previous measurements have shown that reference proteins such as CD86 and CTLA4 can serve as monomeric and dimeric reference proteins to determine the photophysical parameters directly in cells (Fricke et al., 2015; Baldering et al., 2019b). We generated CD86-mEos4b and CTLA4-mEos4b plasmids, transfected these plasmids into HEK293T cells, recorded PALM images, and performed qPALM analysis. We found a p-value of 0.27 for CD86-mEos4b (Figures S2D and S2E), which is equal to that of CD86-mEos3.2 (Baldering et al., 2019b). Next, we used this p-value to analyze the detection efficiency of mEos4b by fitting the CTLA4-mEos4b histogram with a dimeric fit function (Figures S2F and S2G). We obtained a *q*-value of 0.35, which is slightly lower compared to that of CTLA4-mEos3.2 (*q* = 0.39) (Baldering et al., 2019b). A *q*-value of 0.35 translates into a detection efficiency of 79% (see Equation 1 in Supplemental Information). To additionally include the fraction of unlabeled MET receptors, we multiplied the detection efficiency (0.79) with the labeling efficiency (0.81) and obtain an absolute detection efficiency (0.64) that corrects for all undetected MET receptors. We used these parameters (p = 0.27; *d* = 0.64) to analyze the oligomerization of MET-mEos4b in its resting state and found 5%±1% of MET-mEos4b to be dimeric (Figures 2A and S2H). Upon addition of 1 nM HGF, MET-mEos4b showed a dimeric fraction of 71±3% (Figures 2B and S2I). This shows an explicit shift to dimeric MET in the ligand-stimulated state in agreement with the activation model of MET (Comoglio et al., 2008). In an additional experiment, we treated cells with 5 nM of the bacterial ligand InlB_321_-ATTO 647N (Figure S3A). By analyzing receptor clusters on the membrane, we obtained 63±2% dimers, slightly lower compared to the activation of MET with 1 nM HGF (Figure S3B). Additionally, we analyzed receptors only when co-localizing with InlB_321_-ATTO 647N, assuming that these co-localizations show receptors that are bound to InlB_321_-ATTO 647N. The analysis of co-localized spots showed a slightly increased dimer fraction of 68±4% (Figure S3C). These results confirm the ligand-stimulated dimerization of receptor tyrosine kinases.

### Single-particle tracking reveals reduced mobility and immobilization of MET receptors upon ligand stimulation

**Figure 3:**
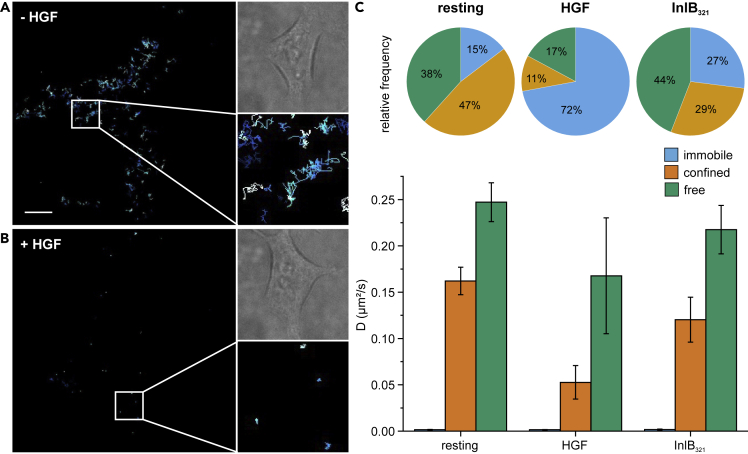
Live-cell single-particle tracking PALM of ligand-stimulated and resting MET-mEos4b SPT analysis of MET-mEos4b in resting (A) and HGF-stimulated (B) cells. The brightfield image and a zoom-in are shown on the right side. Scale bar represents 5 μm. (C) MSD-based diffusion type analysis of resting, HGF-stimulated, and InlB_321_-ATTO 647N-stimulated MET-mEos4b cells. Diffusion coefficients with their standard error of the mean (bar diagram) and the respective fractions (pie charts) of the diffusion states: immobile (blue), confined (orange), and free (green) are shown in their respective color. See also Figure S4.

Receptor mobility was found to respond to ligand binding for various receptor tyrosine kinases (Schlessinger, 2002; Ibach et al., 2015; Harwardt et al., 2020). Therefore, we expected a reduced receptor mobility and an increased fraction of immobile receptors upon stimulation of MET with its physiological ligand HGF or the bacterial ligand InlB_321_. First, we performed live-cell SPT PALM (sptPALM) (Manley et al., 2008) of MET-mEos4b by observing the dynamics of MET receptors. In the resting state, MET-mEos4b was mostly freely diffusing in the membrane of HEK293T cells; only a small fraction of immobile receptors was observed (Figure 3A). In contrast, HGF-stimulated cells showed a strong decrease in free diffusion (Figure 3B). Considerably, more receptors were immobilized showing small diffusion coefficients. To obtain information on diffusion types, we analyzed the trajectories using a mean square displacement (MSD) analysis (Rossier et al., 2012). In resting cells, the relative frequency of the immobile fraction was 15±2% (Figure 3C). sptPALM measurements of MET-mEos4b with 1 nM InlB_321_-ATTO 647N approximately doubled the immobile fraction to 27±3%. Stimulation with 1 nM of the physiological ligand HGF showed a large immobile fraction of 72±3%. Additionally, we show that the diffusion coefficients of the confined and free states decrease with the addition of ligands (Figure 3C). These results demonstrate the sensitivity of MET-mEos4b to its natural ligands and prove the ligand-stimulated immobilization of receptors.

Finally, we performed live-cell universal PAINT (uPAINT) (Giannone et al., 2010) measurements of MET-mEos4b treated with InlB_321_-ATTO 647N by detecting the signal of ATTO 647N, as described recently (Rossier et al., 2012; Harwardt et al., 2017). This enabled us to compare the dynamics of labeled InlB_321_ with mEos4b fused to the MET receptor (Figure S4). The MSD analysis of MET-mEos4b showed lower fractions of immobile receptors (27±3%) compared to InlB_321_-ATTO 647N (44±3%), and diffusion coefficients of InlB_321_-ATTO647N were smaller for confined and free diffusion.

In summary, these results indicate that endogenous tagging by CRISPR/Cas12a in combination with quantitative super-resolution imaging and SPT is a powerful tool to address the molecular organization and dynamics of membrane receptors at an endogenous expression level.

## Discussion

**Figure 4:**
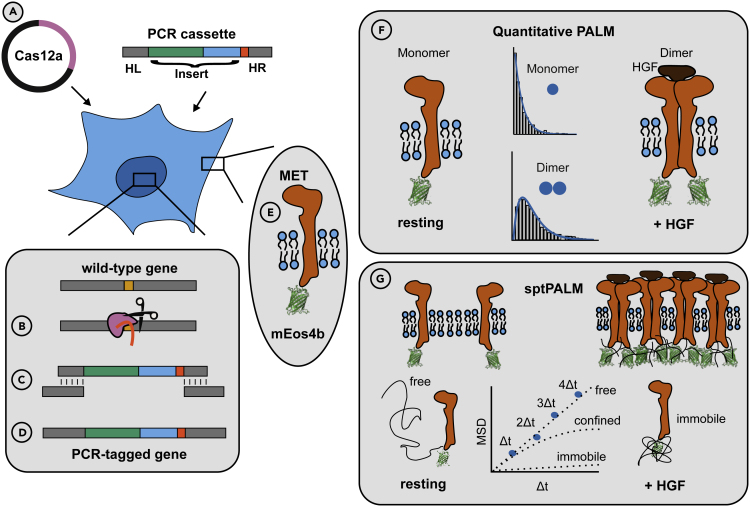
Schematic overview of PCR tagging and possible applications in combination with SMLM (A) Transfection of HEK293T cells with the Cas12a helper plasmid and the individual PCR cassette. (B) Cas12a (pink) cuts the DNA at the target site. (C) The PCR cassette anneals with its homology regions on the genomic DNA and repairs the genomic DNA by homology directed repair (HDR). (D) Illustration of the PCR-tagged gene. (E) The protein of interest is expressed as fusion protein with the tag, e.g., a fluorescent protein, here mEos4b (structure of mEos2 shown; PDB:3S05). (F) qPALM analysis of the PCR-tagged protein of interest reveals its oligomeric state. The MET receptor shows an increased population of dimers in HGF-stimulated cells. (G) Mobility analysis of the PCR-tagged protein of interest with sptPALM. Schematic illustration of MET receptors shows slowdown and immobilization upon HGF stimulation.

The generation of stable cell lines, that express fluorophore-labeled proteins at an endogenous expression level is time intense and often yields poor labeling efficiencies. CRISPR/Cas9 in combination with PALM was used in some studies to label intracellular proteins. The CRISPR/Cas9 method requires (i) a crRNA and tracrRNA expression vector and (ii) cloning of the donor template for homologous insertion of the protein tag. The generation of crRNA expression vectors is usually straightforward, however, requires up to one week for the complete verification of the generated vector. In addition, the synthesis of a donor template by, e.g., Gibson assembly, requires several assemblies to combine all important fragments such as the homologous regions, the tag and an antibiotic resistance (Khan et al., 2017). In comparison, PCR tagging requires less than one day to generate the necessary tools (PCR cassette) for transfection. Since CRISPR/Cas12a is used for PCR tagging, no crRNA expression vector is required as the crRNA is expressed by the PCR cassette itself. Additionally, complicated Gibson assembly reactions are replaced by a simple PCR with primers that can be easily designed. Therefore, PCR tagging enables straightforward and time-efficient gene editing at the C-terminus of proteins of interest (Füller et al., 2020), making it a promising labeling tool for super-resolution microscopy (Figure 4).

We successfully generated a stable cell line expressing MET-mEos4b (Figures 4A–4E). This process was straight forward and time-efficient and will be easily transferable to other targets as previously shown (Füller et al., 2020). We showed the correct insertion of mEos4b into the genome, as well as its homogeneous and endogenous protein expression levels. Füller et al. (2020) reported that occasional off-target integrands seem to result only from spontaneous integrations and that the presence of Cas12a does not promote specific off-target integrands but only on-target integrands (Füller et al., 2020). Since the authors did not observe that such off-target integrations lead to the expression of aberrant fusion proteins, together with our data showing fluorescence emission originating from the plasma membrane but not the cytosol, it is very likely that only the MET receptor effectively expresses mEos4b as a fusion protein.

Further, we verified the activity of the MET-mEos4b fusion protein by western blots. To highlight the various benefits of PCR tagging, we analyzed endogenously tagged MET as transient transfection can induce artifacts due to non-native expression levels (Lisenbee et al., 2003; Doyon et al., 2011; Gibson et al., 2013). We showed the endogenous expression by analyzing the cluster density (cluster/μm^2^) of MET-mEos4b in HEK293T cells. Higher cluster numbers were reported for endogenous MET in HeLa cells in DNA-PAINT measurements (Harwardt et al., 2020). However, taking into account the different mRNA expression levels of MET in HeLa and HEK293T cells (the Human Protein Atlas; www.proteinatlas.org), we expect a lower receptor cluster density for HEK293T cells, which fits to our PALM data and supports an endogenous expression level.

Besides endogenous expression, high labeling efficiency is also important for quantitative analysis. In particular, achieving a high labeling efficiency can be very demanding since the gene of interest can occur on several chromosomes due to multiple sets of chromosomes. PCR tagging allowed us to establish a cell line expressing MET-mEos4b with a protein labeling efficiency of approximately 81%. We assume that our HEK293T cell line contains three copies of chromosome 7 (containing the MET gene) (Stepanenko et al., 2015), where two chromosomes are tagged and transcribed at a higher rate compared to the untagged chromosome (Lo et al., 2003). We achieved this high efficiency through the usage of Cas12a since it has a high specificity *in vivo* (Kleinstiver et al., 2016) and cuts the DNA away from the Cas12a recognition site. This results in repetitive DNA cuts and thus an increased probability for successful incorporation of the homology template DNA (Zetsche et al., 2015; Moreno-Mateos et al., 2017).

The high tagging efficiency allowed us to quantitatively study the stoichiometry of MET receptor in single signaling complexes. We found monomeric MET receptors in absence of a ligand and strongly increased dimerization in the presence of its natural ligand HGF (Figure 4F). This dimeric shift is in agreement with the generally accepted model of ligand-induced dimerization (Schlessinger, 2002), as well as with recent studies on MET dimerization (Li et al., 2020). For the bacterial ligand InlB_321_, we observed a similar increase in dimerization. In previous single-molecule photobleaching experiments, a lower degree of MET dimerization was observed in HeLa cells (Dietz et al., 2013). In addition to the different cell lines, this difference can be explained by incomplete labeling of receptor dimers with InlB_321_-ATTO 647N, as well as unlabeled InlB_321_. We would therefore like to emphasize the benefit of a high tagging efficiency for quantitative SMLM that is provided by PCR tagging.

We further performed SPT experiments of the tagged MET receptor, which showed ligand-induced reduction in the mobility as well as immobilization of receptors (Figure 4G). The diffusion coefficients of MET-mEos4b in resting and ligand-stimulated conditions are similar to previously published values of endogenous HeLa cells (Harwardt et al., 2017). Both studies show ligand-induced slowdown of MET receptor mobility, which demonstrates the reproducibility of MET dynamics in different cell lines. HGF and InlB_321_ showed differently pronounced effects on MET dynamics, which are consistent with the higher affinity of HGF compared to InlB_321_ (Lokker et al., 1992; Dietz et al., 2013). Additionally, we analyzed the dynamics of MET-mEos4b and labeled InlB_321_ in the same cells, which enabled us to determine the dynamics of total MET receptors and of only stimulated ones. As expected, solely InlB_321_-stimulated receptors showed a higher fraction of immobile receptors. Further, diffusion coefficients of exclusively stimulated receptors were smaller compared with the total number of receptors. Since mEos4b and ATTO 647N are spectrally well separated, this opens the door for two-color SPT by investigating the receptor and its ligand at the same time.

In summary, we demonstrate the simple and time-efficient generation of a PCR-tagged cell line and demonstrate super-resolution imaging and single-protein tracking in live cells (Figure 4). We present endogenous labeling of MET-mEos4b as a proof of principle and confirm biological processes such as ligand-stimulated dimerization and immobilization of receptor tyrosine kinases.

### Limitations of the study

This study demonstrates CRISPR/Cas12a-assisted chromosomal labeling of a plasma membrane receptor with a photoswitchable fluorescent protein for quantitative single-molecule super-resolution microscopy. The protocol for CRISPR/Cas12a-assisted chromosomal labeling used in this work allows C-terminal labeling of proteins with fluorescent reporters. Quantitative PALM operates best for low-number oligomers and in total internal reflection fluorescence (TIRF) imaging mode.

### Resource availability

#### Lead contact

Further information and requests for resources and reagents should be directed to and will be fulfilled by the Lead Contact, Prof. Dr. Mike Heilemann, heileman@chemie.uni-frankfurt.de.

#### Materials availability

Additional information including reasonable requests for materials such as plasmids should be directed to and will be conducted by the Lead Contact.

#### Data and code availability

The data included in this study are available from the Lead Contact on reasonable request.

## Methods

All methods can be found in the accompanying Transparent Methods supplemental file.

## Supplemental information

Document S1. Transparent Methods, Figures S1–S4, and Table S1

## Footnotes

Supplemental Information can be found online at https://doi.org/10.1016/j.isci.2020.101895.
