# Supplementary material for: CRISPR/Cas12a-mediated labeling of MET receptor enables quantitative single-molecule imaging of endogenous protein organization and dynamics
Source: iScience. 2020 Dec 7;24(1):101895. doi: 10.1016/j.isci.2020.101895 (PMC7753144; doi:10.1016/j.isci.2020.101895)
Supplement: Document S1. Transparent Methods, Figures S1–S4, and Table S1 [file mmc1.pdf]

iScience, Volume *24*

Supplemental Information

CRISPR/Cas12a-mediated labeling of MET receptor enables quantitative single-molecule imaging of endogenous protein organization and dynamics

Tim N. Baldering, Christos Karathanasis, Marie-Lena I.E. Harwardt, Petra Freund, Matthias Meurer, Johanna V. Rahm, Michael Knop, Marina S. Dietz, and Mike Heilemann

Supplemental Information

Supplemental Figures

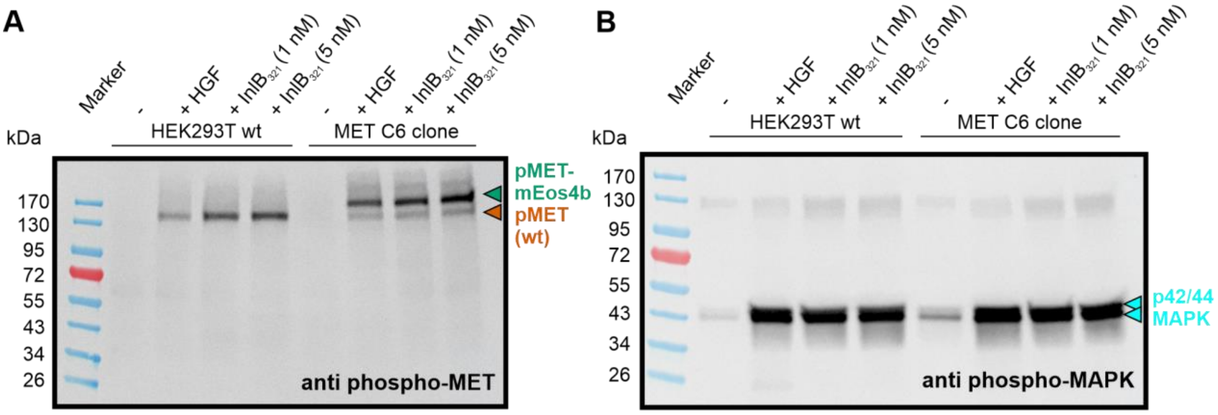

Figure S1. Western blots of ligand-stimulated and resting MET-mEos4b cells. Related to Figure 1. (A) Western blot with an antibody against phospho-MET. Wild-type and MET-mEos4b cells (MET C6) were analyzed under resting conditions as well as upon HGF and $InlB_{321}$-ATTO 647N stimulation. **(B)** Western blot with an antibody against phosphorylated mitogen-activated protein kinase (MAPK) in wild-type (wt) and MET-mEos4b cells (MET C6).

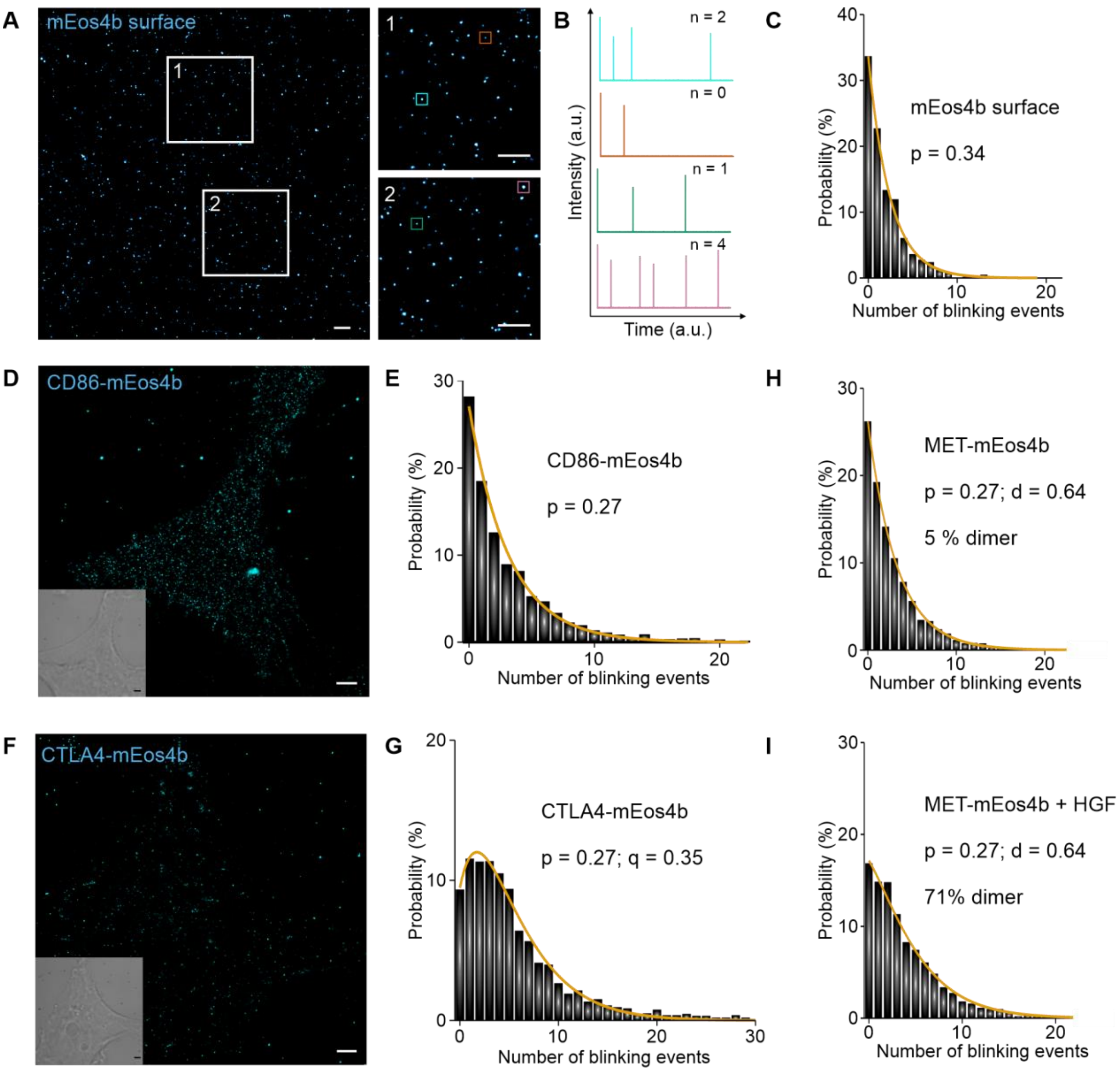

Figure S2. qPALM analysis of single mEos4b molecules as well as resting and HGF-stimulated MET-mEos4b. Related to Figure 2. (A) PALM image of mEos4b on a poly-L-lysine surface with two zoom-ins. Scale bars 2 µm, zoom-ins 1 µm. **(B)** Schematic intensity time traces showing the number of blinking events (n) of single mEos4b molecules. **(C)** Relative frequencies of the number of blinking events of mEos4b molecules (576 clusters). **(D)** PALM image of CD86-mEos4b transfected HEK293T cells. Scale bar is 2 µm. **(E)** Relative frequencies of number of blinking events of CD86-mEos4b molecules (2885 clusters). **(F)** PALM image of CTLA4-mEos4b transfected HEK293T cells. Scale bar is 2 µm. **(G)** Relative frequencies of number of blinking events of CTLA4-mEos4b molecules (2277 clusters). **(H, I)** Relative frequencies of number of blinking events of resting MET-mEos4b (H, 2432 clusters), and HGF-stimulated MET-mEos4b in HEK293T cells (I, 2196 clusters).

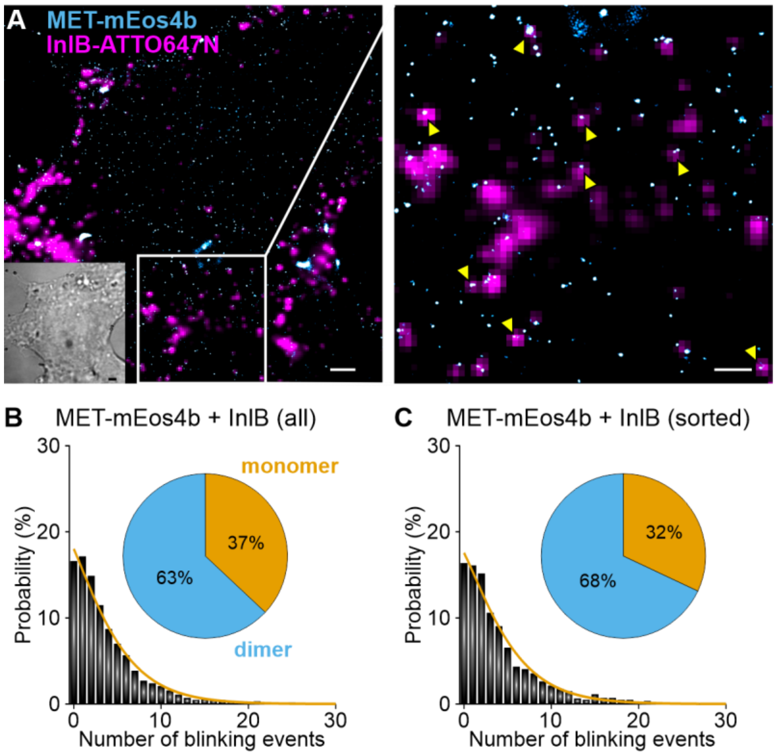

Figure S3. qPALM analysis of InlB$_{321}$-ATTO 647N-stimulated MET-mEos4b cells. Related to Figure 2. (A) qPALM image of MET-mEos4b (cyan) and InlB$_{321}$-ATTO 647N (purple). The brightfield image is shown at the left bottom side. A zoom-in shows the co-localization of MET-mEos4b with InlB$_{321}$-ATTO 647N (yellow arrows). Scale bars 2 µm, zoom-in 1 µm. **(B,C)** Histograms of the relative frequency of blinking events of InlB$_{321}$-ATTO 647N-stimulated MET-mEos4b (B, 3963 clusters) and solely co-localized clusters of MET-mEos4b with InlB$_{321}$-ATTO 647N (C, 1407 clusters).

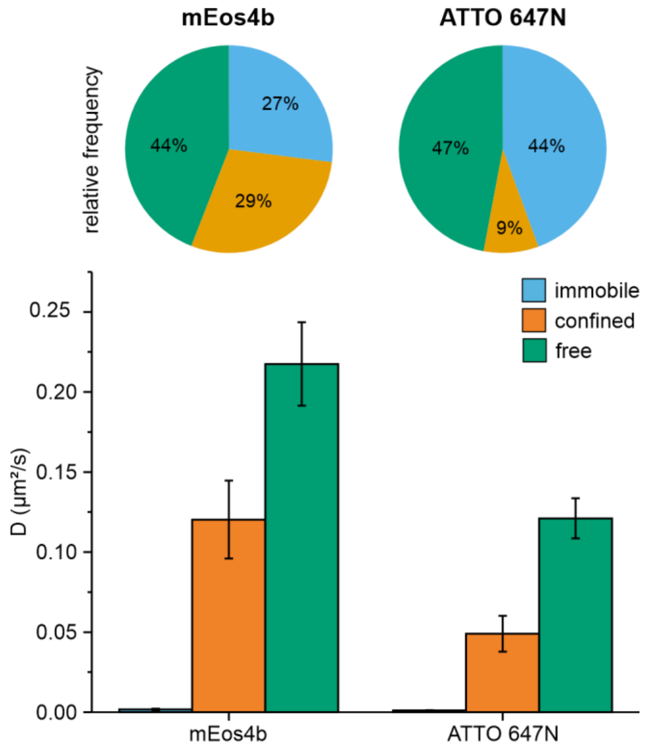

Figure S4. Comparison of live-cell single-particle tracking of MET-mEos4b and $InlB_{321}$-ATTO 647N. Related to Figure 3. MSD analysis of MET-mEos4b (sptPALM) and $InlB_{321}$-ATTO 647N (uPAINT). Diffusion coefficients with their standard error of the mean (bar diagram) and the respective fractions (pie charts) of the diffusion states: immobile (blue); confined (orange), and free (green) are shown in their respective color.

Transparent Methods

Cloning of plasmids

The DNA sequence of mEos4b (pMaCTag-P17, Addgene, plasmid number: #120028) was amplified by PCR (Bio-Rad C1000 Touch™ Thermal Cycler, Bio-Rad laboratories, Hercules, CA, USA) with specific primers (Table 1) and used for cloning into the pRsetA vector for protein expression and purification. The DNA sequence of mEos3.2 (Baldering et al., 2019b) was replaced by mEos4b with standard restriction enzyme cloning and the generated plasmid was verified by DNA sequencing. CD86-mEos4b and CTLA4-mEos4b plasmids were generated in Hot Fusion reactions. PCR fragments were generated with primers (Table 1) containing a 30 base pair overlap, were purified and used in Hot Fusion reactions with 40 ng vector and 200 ng insert in 2x Hot Fusion buffer (0.2 M TRIS HCl pH 7.5, 20 mM $MgCl_2$, 0.4 mM dNTPs, 20 mM DTT, 10% PEG-8000, 0.0075 u/µL T5 exonuclease, and 0.05 u/µL Phusion DNA Polymerase). The Hot Fusion mix was incubated for 1 hour at 50°C, then ramped down to 20°C in 5 minutes (0.1°C per second), and held at 10°C. 3 µL of the Hot Fusion reaction were used for electroporation of *E.coli* Top10 cells. Positive clones were verified by sequencing.

Table S1: List of primers for cloning the plasmids: pRsetA-mEos4b, pirespuro2-CD86-mEos4b and pirespuro2-CTLA4-mEos4b. Related to Figure 2.

Plasmid	Primer name	DNA sequence
mEos4b	mEos4b_fw	GGA GGATCC gtgagtgcgattaagccagacatg
mEos4b	mEos4b_rev	GGA GAATTC tcatcgtctggcattgtcaggc
CD86/CTLA4-mEos4b	Hot_mEos4b_fw	gctgcgctgctggcaaccccggtcgctact gtgagtgcgattaagccagacatgaggatc
CD86/CTLA4-mEos4b	Hot_mEos4b_rev	cacactggatcagttatctatgcggccgct tcatcgtctggcattgtcaggcaatccaga
CD86/CTLA4-mEos4b	Hot_vector_fw	tctggattgcctgacaatgccagacgatga agcggccgcatagataactgatccagtgtg
CD86/CTLA4-mEos4b	Hot_vector_rev	gatcctcatgtctggcttaatcgcactcac agtagcgaccggggttgccagcagcgcagc

Expression and purification of mEos4b

The pRSET vector containing the mEos4b sequence was electroporated (BTX Harvard Apparatus, Gemini System, Thermo Fisher Scientific, MA, USA) into *E.coli* BL21-DE3 cells. Single colonies were picked from a fresh agar plate of electroporated cells and cultivated in 10 mL LB medium (Roth, Karlsruhe, Germany) with the appropriate antibiotic. After about 16 h, 400 mL LB medium was inoculated with 10 mL of the pre-culture and incubated at 37 °C, 200 rpm until the cells reached an OD_{600} (Nanophotometer, IMPLEN, Munich, Germany) of about 0.4. Then, 1 mM isopropyl-ß-D-thiogalactopyranosid (IPTG, Cayman chemical company, MI, USA) was added to induce protein expression for about 16 h at 20 °C, 200 rpm. Then cells were harvested by centrifugation at 4,000 x g for 10 min (Megafuge 1.0, Heraeus, Hanau, Germany). The cell pellet was lysed in 3 mL of lysis buffer (50 mM NaH_2PO_4 (Sigma-Aldrich, MO, USA), 300 mM NaCl (Sigma-Aldrich), 10 mM imidazole (Sigma-Aldrich)) with 1 mM lysozyme (Sigma-Aldrich), a cOmplete Mini EDTA-free protease inhibitor tablet (Roche, Basel, Switzerland) and by 4 x 1 min cycles of sonification (Sonifier 250, Branson Ultrasonics, CT, USA). After lysis, the cell solution was centrifuged two times at 16,900 x g for 15 min (Centrifuge 5418 R, Eppendorf, Hamburg, Germany) and the supernatant was applied onto an equilibrated Ni-NTA column (Qiagen, Venlo, Netherlands). The column was washed with lysis buffer containing 20 mM imidazole and the protein was eluted with lysis buffer containing 250 mM imidazole. Subsequently, the buffer was exchanged to phosphate buffered saline (1xDPBS, Gibco by Thermo Fisher Scientific) by a size-exclusion column. A SDS-PAGE (4-20% gradient SDS gels, BioRad Laboratories, Hercules, USA) analysis verified the correct size of the protein.

Generation of the PCR cassette for endogenous tagging of MET with mEos4b

As a first step of PCR tagging, the PCR cassette was generated (Füller et al., 2020). Primers M1 and M2 (see below) were designed, and a PCR (Bio-Rad C1000 Touch™ Thermal Cycler) was performed with a template plasmid carrying the mEos4b sequence (pMaCTag-P17, Addgene, plasmid number: #120028) (see also: www.PCR-tagging.com). The PCR cassette was purified with a PCR purification kit (Thermo Fisher Scientific GmbH) and further used to transfect HEK293T cells.

Primer M1:
T*G*T*G*T*CGCTCCGTATCCTTCTCTGTTGTCATCAGAAGATAACGCTGATGATGAGGTGGACAC ACGACCAGCCTCCTTCTGGGAGACATCATCAGGTGGAGGAGGTAGTG

Primer M2:
C*A*G*T*G*AAAAAACCATTGGACAAAGTGTGGACTGTTGCTTTGACATAGTACTAGCAAAAAAAAC TAGCACTATGATGTCTCCATCTACACTTAGTAGAAATTAGCTAGCTGCATCGGTACC

Transfection of HEK293T cells and generation of stable CRISPR/Cas12a cell lines
HEK293T cells were cultivated in Dulbecco's modified Eagle medium (DMEM) (Gibco, LifeTechnologies) supplemented with 1% GlutaMAX (Gibco, Life Technologies) and 10% fetal bovine serum (Gibco, Life Technologies) in an CO_2 incubator (Model C 150; Binder GmbH, Tuttlingen, Germany) at 37 °C, 5% CO_2 and seeded on 24-well plates (Greiner Bio-One international GmbH, Kremsmünster, Austria) at a density of $4 \cdot 10^4$ cells/well. After one day, co-transfection with the PCR cassette (500 ng/well in 24 well plate) and the appropriate Cas12a expression plasmid (pY230; Addgene, plasmid number: #89355, 500 ng/well in 24 well plate)) was performed with Lipofectamine 3000 (Thermo Fisher Scientific GmbH) (Füller et al., 2020). After approximately 72 h, the cells were transferred into 6-well plates (Greiner Bio-One international GmbH) containing 1.5 µg/mL puromycin (Sigma-Aldrich). The cells were cultivated in medium containing puromycin for approximately 1-2 weeks to reach a density that was suitable for transferring the cells into a t75 flask (Greiner Bio-One international GmbH). Single clones were then selected using a 96-well plate (Thermo Fisher Scientific) containing 1 cell/well. After an additional week, cells from single wells, containing enough cells from solely one clone, were transferred into 1 well per 24 well-plate and cultivated to have enough cells for genome isolation, western blotting, and analysis through fluorescence microscopy.

Genome isolation and analysis of single clones
Genome isolation was performed with approximately $1 \cdot 10^6$ cells by using the Genomic DNA Purification Kit (Thermo Fisher Scientific GmbH). After genome isolation, a PCR was performed with primers annealing in- and outside of the PCR cassette. Fragments were purified and sent to Eurofins genomics for DNA sequence analysis.

Western blot of single clones
Individual clones were seeded on 10 cm dishes at a density of about $1 \cdot 10^6$ cells per dish. After 2.5 days, cell medium was changed to serum-free medium for approximately 12-16 h. Cells were stimulated with 1 nM human HGF (PeproTech GmbH, Hamburg, Germany) and 1 or 5 nM $InlB_{321}$-ATTO 647N for 10 minutes at 37 °C and then washed with PBS once prior to harvesting the cells with 200 µL of cell lysis buffer (50 mM Tris (Sigma-Aldrich), 150 mM NaCl (Sigma-Aldrich), 1% Triton X-100 (Sigma-Aldrich), 1 mM Na_3VO_4 (Sigma-Aldrich), 1mM EDTA (Sigma-Aldrich), 1mM NaF (Sigma-Aldrich), and one-fourth of a cOmplete Mini EDTA-free protease inhibitor tablet (Roche)). After the cells were incubated in a temperature shaker (Thermo-Shaker, Universal Labortechnik GmbH & Co. KG, Leipzig, Germany) at 750 rpm; 4 °C for 5 min, cells were centrifuged at 12,000 x g; 4 °C for 20 min (Centrifuge 5418 R, Eppendorf). The supernatant was collected and supplemented with 5x SDS-loading dye (250 mM Tris-HCl (pH 6.8), 8% (w/v) SDS (Sigma-Aldrich), 0.1% bromphenol blue (Sigma-Aldrich), 40% (v/v) glycerol (Roth)) and a final concentration of 100 mM dithiothreitol (DTT, Sigma-Aldrich). SDS-PAGE was performed with 4-20% gradient SDS gels (BioRad Laboratories) at 200 V for 30-90 min. Gels were blotted with the iBlot Gel Transfer System (Invitrogen, Thermo Fisher Scientific) as described by the manufacturer. After blotting, membranes were transferred into 50 mL tubes and 5% (w/v) nonfat dry milk (Cell Signaling Technology, MA, USA) in Tris-buffered saline containing 0.1% Tween-20 (TBST) was added for 1 h at ambient temperature. The membranes were washed three times with 10 mL TBST and incubated with primary antibody (MET antibody, #4560; phospho-MET antibody (tyr1234/1235), #3077; phospho-MAPK antibody, #9101S; Cell SignalingTechnology, MA, USA, 1:1000) at 4 °C over night. After three additional washing steps with 10 mL TBST, the secondary HRP-tagged antibody (goat anti-rabbit IgG, Jackson ImmunoResearch, PA, USA, 1:20,000) was added for 3 h at ambient temperature. Then, the membrane was washed three times with 10 mL TBST and once with 10 mL TBS for 5-15 min at ambient temperature. Finally, chemiluminescence detection at a Fusion FX Edge imager (Vilber Lourmat, Collégien, France) was performed by using 1 mL of SuperSignal West Femto Maximum Sensitivity Substrate solution (Thermo Fisher Scientific). Western blot bands were analyzed with Fiji

(Schindelin et al., 2012). The integral of the intensity of individual bands was determined, which allowed the fraction of labeled MET receptor to be estimated.

Preparation of functionalized surfaces for SPT and qPALM

Before sample preparation, the cover glass passivation and functionalization was performed (Baldering et al., 2019b). 25 mm round glass coverslips (VWR International, Radnor, USA) or square cover glasses (35 × 64 mm, # 1.5, Thermo Fisher Scientific) were sonicated in 2-propanol (VWR Chemicals) for 20 min, plasmacleaned with nitrogen or oxygen for 15 min (Diener Electronic GmbH, Ebhausen, Germany) and covered with 100 µg/mL of poly-L-lysine (Sigma-Aldrich) or 0.8 mg/mL PLL-PEG-RGD (self-made, for details see (Harwardt et al., 2020)) for 2 h. After washing with H_2O, cover glasses were dried with nitrogen and used immediately or stored under argon gas at -20 °C.

Sample preparation of mEos4b and MET-mEos4b for (q)PALM imaging

The purified mEos4b protein was transferred on a poly-L-lysine coated cover glass (35 × 64 mm, #1.5, Thermo Fisher Scientific) using 8-well flexiPERMs (Sarstedt) at a concentration suitable for PALM imaging (about 1 nM) and incubated for 30 min at ambient temperature. After that, the chambers were washed three times with sterile-filtered PBS and PALM movies were recorded in sterile-filtered PBS.

200 ng plasmid of CD86-mEos4b and CTLA4-mEos4b were transfected into HEK293T cells in 6-well plates at ~60% confluence with Lipofectamine 3000 (Thermo Fisher Scientific) according to the manufacturer's instructions. After about 24 h, the cells were transferred on PLL-PEG-RGD coated cover glasses (35 × 64 mm, # 1.5, Thermo Fisher Scientific) using flexiPERM chambers. After about 16 h, cells were washed with 400 mM saccharose (Sigma-Aldrich) in PBS and fixed with 4% paraformaldehyde (Thermo Fisher Scientific), 0.1% glutaraldehyde (Sigma-Aldrich) and 400 mM saccharose in PBS buffer for 15 minutes. Then, cells were washed three times with sterile-filtered PBS. Gold particles (120 nm; Nanopartz, Loveland, CO, USA) were sonicated for 10 minutes and added in a 1:5 dilution in PBS to the cells for 8 minutes. Cells were washed again three times with PBS and PALM movies were recorded in sterile-filtered PBS.

MET-mEos4b cells were seeded on PLL-PEG-RGD coated cover glasses (35 × 64 mm, # 1.5, Thermo Fisher Scientific) at a density of $(2-2.5)\cdot 10^4$ cells/well using 8-well flexiPERMs (Sarstedt, Nümbrecht, Germany). After approximately 16-24 h, the cells were washed once with serum-free imaging medium containing DMEM with 1% GlutaMAX and 50 mM HEPES buffer (pH 7.2–7.5; Gibco, Life Technologies, CA, USA) and incubated in serum-free imaging medium for approximately 4 hours. Afterwards, cells were incubated at 4° C for 5 min. Either HGF, $InlB_{321}$-ATTO 647N or no ligand was added to the cells in concentrations of 1 nM or 5 nM, respectively, for 10 minutes at 4° C. After that, cells were washed with 400 mM saccharose (Sigma-Aldrich) and fixed with 4% paraformaldehyde (Thermo Fisher Scientific), 0.1% glutaraldehyde (Sigma-Aldrich) and 400 mM saccharose in PBS buffer for at least 15 minutes. Then, cells were washed three times with sterile-filtered PBS. Gold particles (120 nm; Nanopartz, Loveland, CO, USA) were sonicated for 10 minutes and added in a 1:4 solution in PBS to the cells for 10 minutes. Cells were washed again three times with PBS and PALM movies were recorded in sterile-filtered PBS.

(q)PALM imaging of mEos4b, CD86-mEos4b, CTLA4-mEos4b and MET-mEos4b

Quantitative PALM movies were recorded using a custom-built widefield setup equipped with an inverted microscope (Olympus IX71). The microscope was equipped with an 100x oil immersion objective (PlanApo 100 × TIRFM, NA≥1.45, Olympus) and a nose piece for drift minimization. Lasers with the wavelength of 405 nm (LBX-405-50-CSB-PP, Oxxius, 0–30 mW/cm^2), 568 nm (Sapphire 568 LP, Coherent, 0.21 kW/cm^2) and 638 nm (LBX-638-180, Oxxius, 4.6 W/cm^2) were coupled into the objective and movies were recorded with total internal reflection (TIR) illumination. Bandpass filters (ET 700/75; BrightLine HC 590/20, AHF) were used to filter the emission light. SMLM movies of 15,000–40,000 frames were recorded with an EMCCD camera (iXon Ultra, Andor) with a physical pixel size of 157 nm (camera pixel and magnification), an exposure time of 100 ms, and an EM gain of 200 until almost no blinking was observed. For each condition a minimum of ten cells from at least three different measuring days were used for data analysis.

(q)PALM data analysis

Single-molecule localizations were filtered using rapidSTORM (v.3.3) (Wolter et al., 2010)Klicken oder tippen Sie hier, um Text einzugeben.. The super-resolved images were loaded into Fiji (Schindelin et al., 2012) and cell areas were determined. Single clusters per cell were analyzed with the 3D Object counter v2.0 (Bolte and Cordelières, 2006) with a threshold of 125 and a minimum size filter of 10. Receptor densities were obtained from 21 cells by dividing the total number of clusters by the respective cell area. The error is given as standard deviation.

Detailed protocols for the data analysis of qPALM movies have been presented (Krüger et al., 2017; Baldering et al., 2019b; Karathanasis et al., 2020). Briefly, PALM images were generated with rapidSTORM (v3.3) and localizations of consecutive frames were grouped with a distance threshold of 90 nm. Furthermore, LocAlization Microscopy Analyzer (LAMA) (Malkusch and Heilemann, 2016) was used to extract the number of blinking events of single localization clusters in the super-resolved images. Since individual cells do not contain enough statistics, we randomly mixed the data of each condition ten times and analyzed 80 percent of the respective data. Histograms of the relative frequency of number of blinking events were plotted and fitted with the qSMLM software (Baldering et al., 2019a) yielding the corrected monomer/dimer fractions. The mean values were determined for each condition and the error is given as standard deviation.

The q-value obtained from the blinking histogram of CTLA4-mEos4b was converted into the detection efficiency (d) according to the following equation (1):

$$d = \frac{2-2q}{2-q} \quad (1)$$

SPT sample preparation and imaging of MET-mEos4b

MET-mEos4b cells were seeded on PLL-PEG-RGD coated round cover glasses at a density of $(20 - 30) \cdot 10^4$ cells/well in 6-well plates. After approximately 24 h, the round cover glasses were transferred into custom-built holders. The cells were washed once with serum-free imaging medium containing DMEM with 1% GlutaMAX and 50 mM HEPES buffer (pH 7.2–7.5; Gibco, Life Technologies). In the presence of ligands, either HGF or $InlB_{321}$-ATTO647N was added to the cells at a concentration of 1 nM in imaging medium. sptPALM and uPAINT measurements were performed using a commercial microscope (N-STORM, Nikon). The microscope was equipped with a 100x objective (100 × Apo TIRF oil, 1.49 NA), a 647 nm laser (0.2 kW/cm^2), a 561 nm laser (0.2 kW/cm^2), a 405 nm laser (0-38 mW/cm^2) and was operated with TIR illumination. Image acquisition was controlled by µManager (version 1.4.20) (Edelstein et al., 2014) and NIS-Elements (version 4.30.02, Nikon, Düsseldorf, Germany) using an exposure time of 20 ms and an EM gain of 300. sptPALM or uPAINT movies were recorded with an EMCCD camera (DU-897U-CS0-BV; Andor Technology, Belfast, UK), using a physical pixel size (camera and magnification) of 158 nm. Recorded movies had a length of 1,000 frames. For each condition about 20 cells from at least three different days were used for data analysis.

SPT data analysis

The data analysis of sptPALM measurements was performed with a plugin for MetaMorph (version 7.7.0.0, Molecular Devices, Sunnyvale, USA), called PALM-Tracer (Bordeaux Imaging Center, France) (see also (Harwardt et al., 2017)). Localizations were determined by centroid fitting and connected into trajectories if the distance between two subsequent localizations did not exceed 5 pixels (790 nm). MSD values were calculated from these trajectories and diffusion coefficients were determined by fitting the first four points of the MSD plot. MSD data were also used in diffusion type analysis. Based on the dynamic localization precision the smallest determinable diffusion coefficient D_{min} (0.008 µm²/s) was calculated and all diffusion coefficients $d \leq D_{min}$ were assigned as immobile. Further analyses (Rossier et al., 2012) were used to differentiate between confined and free diffusion applying a τ value of 60 ms as threshold. Statistical analysis of diffusion coefficients and diffusion types was performed in Origin (OriginPro 2016G, OriginLab).

Supplemental References

Bolte S, Cordelières FP (2006). A guided tour into subcellular colocalization analysis in light microscopy. J Microsc 224, 213–232.

Edelstein AD, Tsuchida MA, Amodaj N, Pinkard H, Vale RD, Stuurman N (2014). Advanced methods of microscope control using μManager software. J Biol Methods 1.

Krüger C, Fricke F, Karathanasis C, Dietz MS, Malkusch S, Hummer G, Heilemann M (2017). Molecular counting of membrane receptor subunits with single-molecule localization microscopy. In: Jörg Enderlein, Ingo Gregor, Zygmunt K. Gryczynski, Rainer Erdmann und Felix Koberling (Hg.). SPIE BiOS. San Francisco, California, United States, Saturday 28 January 2017: SPIE (SPIE Proceedings), 100710K.

Malkusch S, Heilemann M (2016). Extracting quantitative information from single-molecule super-resolution imaging data with LAMA - LocAlization Microscopy Analyzer. Sci Rep 6, 34486.

Schindelin J, Arganda-Carreras I, Frise E, Kaynig V, Longair M, Pietzsch T, Preibisch S, Rueden C, Saalfeld S, Schmid B, Tinevez J-Y, White DJ, Hartenstein V, Eliceiri K, Tomancak P, Cardona A (2012). Fiji. An open-source platform for biological-image analysis. Nat Methods 9, 676–682.

Wolter S, Schüttpelz M, Tscherepanow M, van de Linde S, Heilemann M, Sauer M (2010). Real-time computation of subdiffraction-resolution fluorescence images. J Microsc 237, 12–22.
